# Vitellogenins in the spider *Parasteatoda tepidariorum* – expression profile and putative hormonal regulation of vitellogenesis

**DOI:** 10.1186/s12861-019-0184-x

**Published:** 2019-03-08

**Authors:** Agata W. Bednarek, Marta K. Sawadro, Łukasz Nicewicz, Agnieszka I. Babczyńska

**Affiliations:** Department of Animal Physiology and Ecotoxicology, University of Silesia, Bankowa 9, PL40007 Katowice, Poland

**Keywords:** Vitellogenesis, Gene expression, Spiders, Hormonal regulation, 20-hydroxyecdysone, Fenoxycarb, Precocene, Juvenile hormone

## Abstract

**Background:**

Knowledge about vitellogenesis in spiders is rudimentary. Therefore, the aim of study was to check the vitellogenin (Vg) presence in various tissues of the female spider *Parasteatoda tepidariorum*, determine when and where vitellogenesis starts and takes place, and the putative role of selected hormones in the vitellogenesis.

**Results:**

Here we show two genes encoding Vg (Pt*Vg*4 and Pt*Vg*6) in the genome of the spider *P. tepidariorum*. One gene Pt*Vg*4 and three subunits of Vg (250 kDa, 47 kDa and 30 kDa) are expressed in the midgut glands, ovaries and hemolymph*.* Heterosynthesis of the Vg in the midgut glands and autosynthesis in the ovaries were observed. Vitellogenesis begins in the last nymphal stage in the midgut glands (heterosynthesis). However, after sexual maturity is reached, Vg is also synthesized in the ovaries (autosynthesis). Changes in the Pt*Vg*4 expression level and in the Vg concentration after treatment with 20-hydroxyecdysone, a juvenile hormone analog (fenoxycarb) and an antijuvenoid compound (precocene I) were observed. Therefore, we propose a hypothetical model for the hormonal regulation of vitellogenesis in *P. tepidariorum*.

**Conclusions:**

Our results are the first comprehensive study on spider vitellogenesis. In our opinion, this work will open discussion on the evolutionary context of possible similarities in the hormonal control of vitellogenesis between *P. tepidariorum* and other arthropods as well as their consequences.

## Background

Vitellogenesis is one of the most important processes that enable proper and efficient reproduction in animals [1]. It consists of the synthesis and accumulation of proteins (yolk) that are the source of energy for a developing embryo in oviparous animals. Among arthropods, the course of the vitellogenesis is well known in insects [2]. The main compound of the yolk is vitellin (Vn), which is produced as a result of crystallization processing of the precursor – vitellogenin (Vg, plural – Vgs). Insect vitellogenesis consists of several stages: (i) synthesis of Vg at the transcript level (transcriptional and post-transcriptional modification) with translational and post-translational modifications (ii) secretion of these proteins into the hemolymph and their transport to the ovaries, and (iii) uptake of Vgs by the ovaries and incorporation of proteins in the developing oocytes [2, 3]. A similar course of vitellogenesis is demonstrated in ticks (e.g. [4–7]). Each stage is subject to strict neurohormonal control. Essential compounds that are involved in the regulation of vitellogenesis in arthropods are: juvenile hormones, ecdysteroids and neuropeptides [8–20].

Knowledge about vitellogenesis in spiders is rudimentary despite the fact that the first publication on this topic was 61 years ago [21, 22]. This knowledge is primarily based on the histological analysis of ovaries [21–25]. These studies about *Eratigena atrica* and *Tegenaria domestica* indicate that ovaries in the pre-vitellogenic phase are observed in the virgin females while mated females contain ovaries in vitellogenesis and post-vitellogenesis phases. However, recently Guo et al. [26] have presented the first study about the Vg transcript level in spider *Pardosa pseudoannulata*.

As yet, when vitellogenesis starts in spiders is still not known for certain, although it appears may be species-specific [25–27]. The site for the synthesis of Vgs is also puzzling. In insects, Vgs are produced in the fat body. This tissue is also responsible for the Vg synthesis in ticks – spiders’ closest arthropod relatives. Moreover, in ticks Vg synthesis takes place in the midgut (heterosynthesis [7, 28, 29] and perhaps in the fat body (heterosynthesis [30], possibly in the ovaries (autosynthesis [6]) and in the pedicel cells (third source of the Vg heterosynthesis; [4, 5]). Because the fat body does not exist in spiders [31], another organ must be responsible for Vg synthesis in this group of arthropods.

Knowledge about the hormonal control of vitellogenesis is also undeveloped and unclear in spiders. It should be emphasized that data about the presence and general role of juvenile hormone and ecdysteroids in spiders are scarce (compare with [32]). However, it appears that ecdysteroids and juvenile hormone exist in spiders, at least in the spider *Parasteatoda tepidariorum*. Our preliminary studies revealed the presence of genes encoding enzymes of the biosynthesis pathway of ecdysteroids and juvenile hormone and receptors of these hormones in *P. tepidariorum* (unpublished data). Furthermore, the presence of ecdysteroid receptors and ecdysteroids has been reported in the spider *Agelena silvatica*. These proteins are functional ecdysteroid receptors, at least during the moulting process of this species of spider [33]. Moreover, several authors believe that ecdysteroids (and probably also juvenile hormones) may play the main role in regulating the synthesis of the Vgs in spiders [24, 34, 35]. On the other hand, Stubbendieck et al. [36] indicated a potential lack of the role of ecdysteroids in the initiation of vitellogenesis at least in spiders of the *Schizocosa genus*. Neese et al. [37] and Connat et al. [38] on ticks show that ecdysteroids and juvenile hormone (if exist in this group of arthropods) play a role in the regulation of vitellogenesis and oviposition processes [12, 15, 39–42]. Thus, it seems that these hormones can perform similar functions in the close relative spiders.

The Vg at the gene and protein levels is still not well described in spiders. Recently Guo et al. [26] have shown that there are three genes encoding Vg in wolf spider *P. pseudoannulata*. Their expression pattern is dependent on the spider developmental stages and is connected with mating and egg laying. However, the site of Vg gene expression is still not known. Moreover, the lack of data about the number of subunits for Vgs and profile of their concentration during development is observed.

Here we analyzed the Vg presence at transcript and protein levels in various tissues of the female spider *P. tepidariorum* and their fluctuation during different stages of the spider development. Moreover, we tested the role of hormones (ecdysteroids and the juvenile hormone) in the regulation of vitellogenesis in *P. tepidariorum* by bioassays based on the application of hormones and substances with (anti) hormonal activity.

## Results

### Identification of Pt*Vg4* and Pt*Vg6*, the sex and tissue source of Pt*Vg4* and analysis of Pt*Vg4* expression

In silico analysis of the genome of *P. tepidariorum* identified two genes that encode the putative vitellogenin. These sequences were identical to sequences from the NCBI Protein databases [43] (the vitellogenin-4-like isoform X1 of *P. tepidariorum*, accession no. XP_015930209 and the vitellogenin-6 *P. tepidariorum*, accession no. XP_015930207). However, only one (the vitellogenin-4-like isoform X1 – Pt*Vg*4) met the criterion of sequence homology [44]. Therefore, further analysis was only based on the Pt*Vg*4 sequence.

**Fig. 1:**
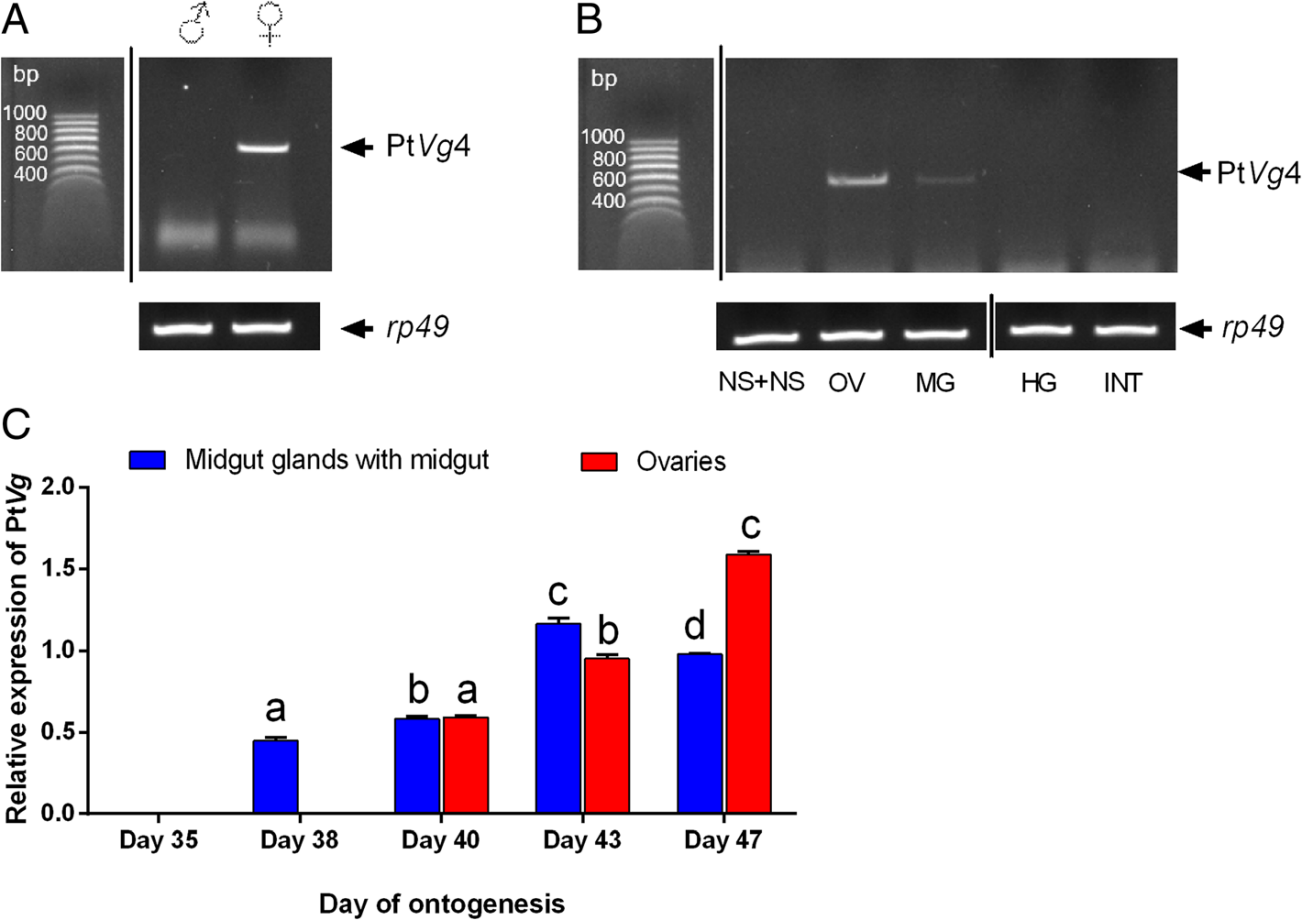
Pt*Vg*4 gene is expressed in the ovaries and midgut glands only of female spiders. PCR and qPCR analyses of the sex- and tissue-specific expression of *P. tepidariorum* Vg (Pt*Vg*4). **a** Sex-specific expression of Vg in the whole body extract on the first day after mating. **b** Tissue-specific expression of the Pt*Vg*4 in female spiders on day 47 after leaving a cocoon. Total RNA samples were extracted from various female tissues: the nervous system and neuroendocrine system (NS + NS), ovaries (OV), the midgut glands (MG), hindgut (HG), integument (INT). **c** Pt*Vg*4 mRNA level [average ± SD] in female spiders in various ages – in the penultimate nymph stage (day 35 after leaving a cocoon), last nymphal stage (day 38), day that sexual maturation was reached (day 40), three days after mating (day 43) and the day of the first oviposition (day 47) in the midgut glands and in the ovaries. Lowercase letters indicate significant differences within the same tissue (Tukey’s multiple comparisons test, *p* ≤ 0.05)

For this part of the study, the whole body extract of 40 male and 350 female spiders were used. The *P. tepidariorum* Vg4 (Pt*Vg*4) was expressed specifically in the whole body extract of females after mating, but not in males (Fig. 1a). The presence of the Pt*Vg*4 transcript was observed in the ovaries and the midgut glands, but not in other tissues, including the nervous system and neuroendocrine system, hindgut and integument (Fig. 1b).

The Pt*Vg*4 levels fluctuated during tested part of the *P. tepidariorum* females development in all of the tested tissues. Presence of the Pt*Vg*4 transcript was observed in the midgut glands beginning on day 38 after leaving a cocoon and after day 40 in the ovaries. Expression of the gene in the midgut glands increased linearly until day 43 after leaving a cocoon. On the other hand, the Pt*Vg*4 levels in the ovaries were characterized by a linear increase (*R*^*2*^ = 0.97) until day 47 after leaving a cocoon. In addition, the Pt*Vg*4 expression showed a very strong positive correlation with the age of spider in both tissues (the midgut glands – *r* = 0.91, *p* = 0.02 and the ovaries – *r* = 0.98, *p* = 0.01).

### Identification of vg subunits and tissue source of vg and analysis of vg concentration

Vitellogenin was distinguished electrophoretically by comparing polypeptide profiles of all two-day-old eggs from 25 cocoons (egg mass of each cocoon was one sample, *n* = 25), the whole body extract of mature males (*n* = 25) and ovaries of 42 females in various ages (determined by the number of days after leaving a cocoon) (for each age – *n* = 6).

**Fig. 2:**
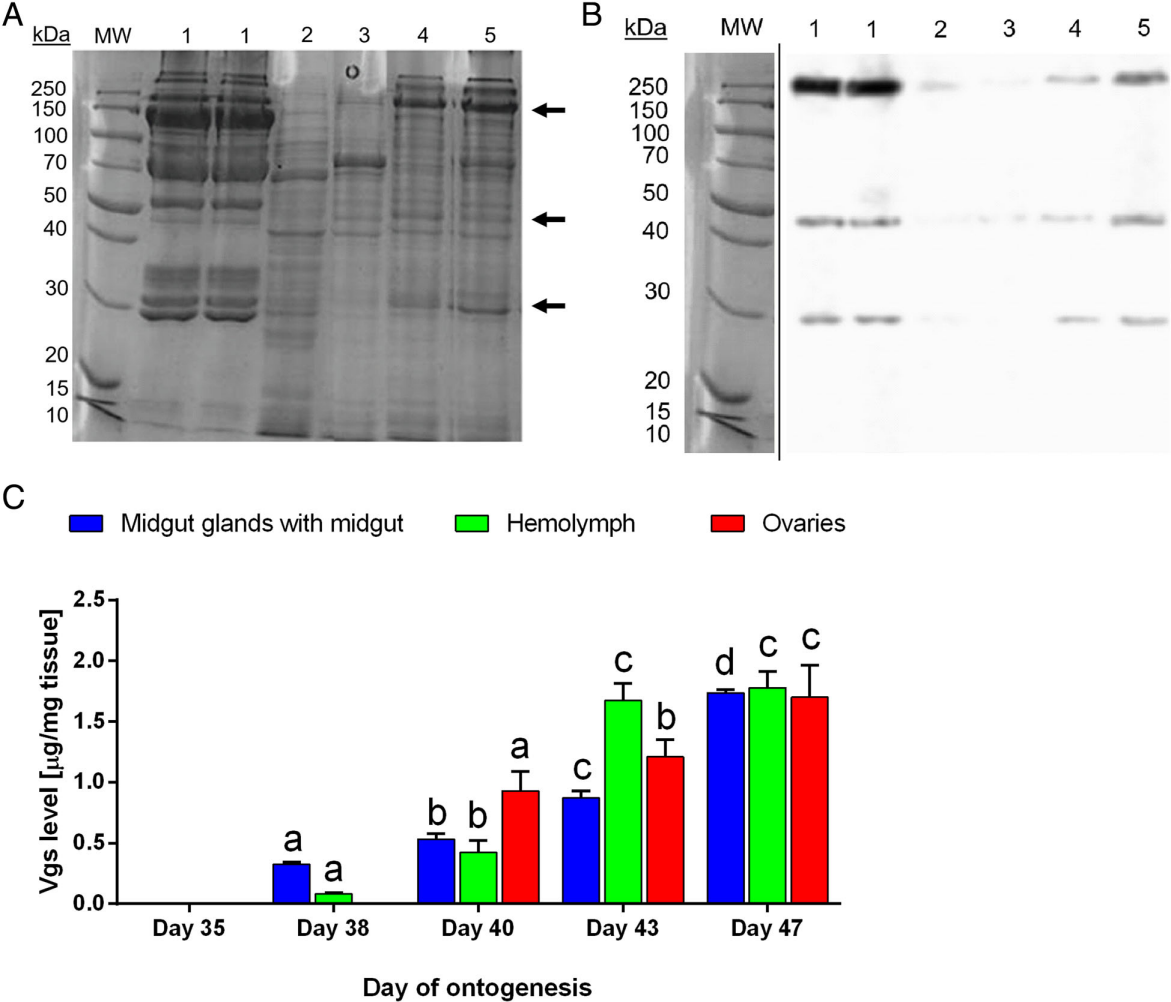
Subunits of the Vg and their fluctuation in the tested part of development of the *P. tepidariorum.* Visualization of three subunits (250 kDa, 47 kDa and 30 kDa) of the Vgs (arrows) in the homogenate from: eggs (1), whole body extract of males after mating (2), ovaries from females on day 38 after leaving a cocoon (3), day 40 after leaving a cocoon (4), day 43 after leaving a cocoon (5) by SDS-PAGE electrophoresis (**a**) and Western blot (**b**). Fluctuations in the Vg proteins [average ± SD] in: (**c**) the midgut glands, hemolymph and ovaries of the female spiders in different ages. Lowercase letters indicate significant differences within the same tissue (Tukey’s multiple comparisons test, *p* ≤ 0.05)

SDS-electrophoretic analysis revealed that three prevailing polypeptides were present in the egg extract (line 1 in Fig. 2a and b), in females in the last nymphal stage (on day 38 after leaving a cocoon, line 3 in Fig. 2a and b) and in the reproductively-active females (virgin females on day 40 after leaving a cocoon and females after mating on day 43 after leaving a cocoon, (lines 4 and 5 in Fig. 2a and b), but were absent (or present only in a small amounts) in homogenates from the whole body extract of males (line 2 in Fig. 2a and b). The average relative molecular weights (MW) of subunits of these putative proteins calculated from measurements (three acrylamide gels) for each sample were: 250.40 ± 1.25 kDa, 47.5 ± 3.1 kDa and 30.02 ± 6.45 kDa. In addition, results were confirmed by analyzing the outcomes from Western blot (three Western-blot membranes; Fig. 2b).

Determining the concentration of Vg in the midgut glands, ovaries and hemolymph was performed using ELISA. Tissues from 156 females were used for this test. ELISA results indicate fluctuation the Vg concentration in all of tested tissues (Fig. 2). The presence of Vg was observed in the midgut glands and hemolymph from the last nymphal stage (day 38 after leaving a cocoon), and in the ovaries from the day of becoming mature and mating (day 40 after leaving a cocoon). In all of the tissues analyzed, Vg was detectable until day 47 (the day the day of the first oviposition). In addition, the level of Vg increased linearly in the midgut glands and ovaries during tested part of the spider development (respectively, *R*^*2*^ = 0.92 and *R*^*2*^ = 0.95). These observations confirm a very strong positive correlation between changes in the Vg concentration and the age of spiders, both in the midgut glands (*r* = 0.991, *p* = 0.001) and ovaries (*r* = 0.989, *p* = 0.001). On the other hand, the concentration of Vg in the hemolymph only increased up to day 43 after leaving a cocoon (Fig. 2c), after which it was stabilized at this level until the end of observations.

### Role of ecdysteroids and juvenile hormone in the regulation of the vitellogenesis

The putative role of 20-hydroxyecdysone, a juvenile hormone analog – fenoxycarb and an antijuvenoid – precocene I was validated in cohorts of the female spiders on day 35 after leaving a cocoon (penultimate nymphal stage) (500 spiders for each dose and time point).

MANOVA analysis of results from each test for each compound indicated that the expression level of Pt*Vg*4 and Vg concentration (in female spiders of *P. tepidariorum*) were statistically significantly dependent on the dose of the compound that was administered (*p* < 0.0001), the type of tissue (*p* < 0.0001) and the time that elapsed after injection/topical applications (*p* < 0.0001).

### Effect of 20E on Pt*Vg*4 expression and vg concentration

**Fig. 3:**
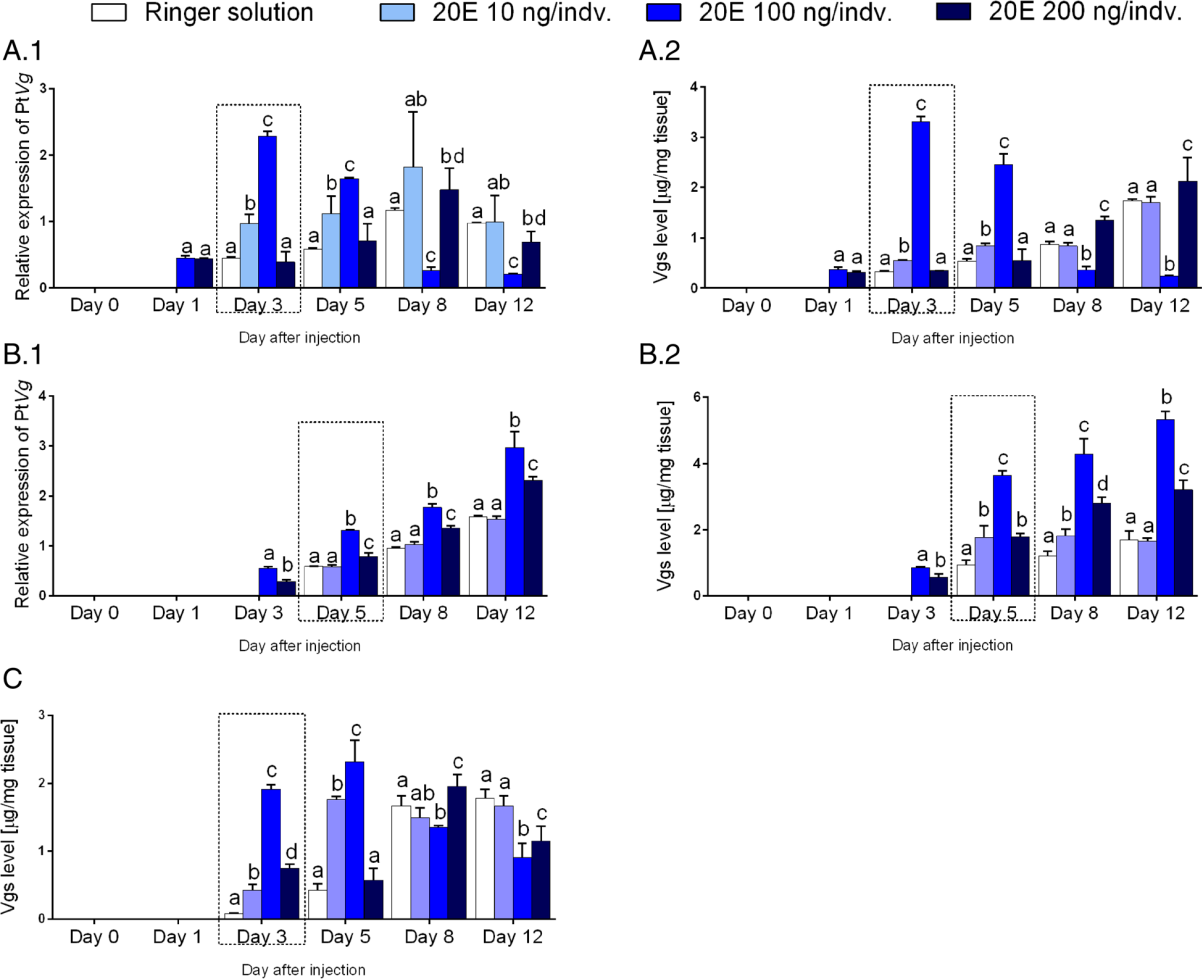
Level of vitellogenin (at transcript and protein levels) in response to 20-hydroxyecdysone administration. The profile **of the vitellogenins** [mean ± SD] of the P. tepidariorum females that were treated with a Ringer solution and 10, 100 and 200 ng of 20E per individual. Vg results in the midgut glands at the transcript (**a**.1) and protein levels (**a**.2), the ovaries at the transcript (**b**.1) and protein levels (**b**.2) and the hemolymph at the protein level (**c**). The dashed line box shows the physiological time of vitellogenesis. Lowercase letters indicate significant differences between experimental groups at the same time point (Tukey’s multiple comparisons test, *p* ≤ 0.05)

Injection of 20E to spiders had a significant effect on the Pt*Vg*4 expression and Vg concentration in tested tissues. The presence of the Pt*Vg*4 transcript and Vg proteins in the midgut glands and ovaries in spiders treated with 20E in doses of 100 and 200 ng/indv 2 days earlier than under physiological conditions in the control group was observed (Fig. 3a and b). What’s more, the Pt*Vg*4 expression level and Vg concentration were much higher in the midgut glands (respectively, 5 and 10-times) and ovaries (respectively, 2.22 and 4-times) after the treatment of the 20E in the most effective dose (it seems that this is the 20E in dose 100 ng/indv) compare with the day when the Pt*Vg*4 expression and the Vg presence is noted for the first time in these tissues in the control group (day 38 after leaving a cocoon, i.e. the day 3 of the experiment in the midgut glands and day 40 after leaving a cocoon, i.e. the day 5 of the experiment in the ovaries).

Interestingly, the most effective dose of the 20E that is responsible for the highest increases of the level of the Pt*Vg*4 expression and Vg concentration in tested tissues, caused a 5-time decrease in the Pt*Vg* transcript level and (Fig 3a1) a 7-time decrease in the Vg concentration (Fig 3a2) respectively, on day 8 and 12 of the experiment, but only in midgut glands and not in the ovaries (Fig. 3b). The level of Vg in the ovaries increased linearly during the entire experiment at the transcript level (only after 100 ng 20E/indv treatment; *R*^*2*^ = 0.98) as well as the protein level (after 100 and 200 ng/indv 20E treatment, *R*^*2*^ = 0.92 and *R*^*2*^ = 0.94, respectively).

The early presence of Vg proteins as in the midgut glands and ovaries was not observed in the hemolymph (Fig. 3c). In the hemolymph, Vg was detected in experimental groups at the same time as in the control group. However, Vg concentration was 24-time higher (after treatment of the 20E in dose 100 ng/indv) on the day of Vg appearance (on 3 day of experiment) in the hemolymph compared with the control group. Interestingly, a 2-time decrease in the Vg concentration was observed on the last day of the experiment after administration of 20E at doses of 100 ng and 200 ng/indv.

### Effect of fenoxycarb on Pt*Vg*4 expression and vg concentration

**Fig. 4:**
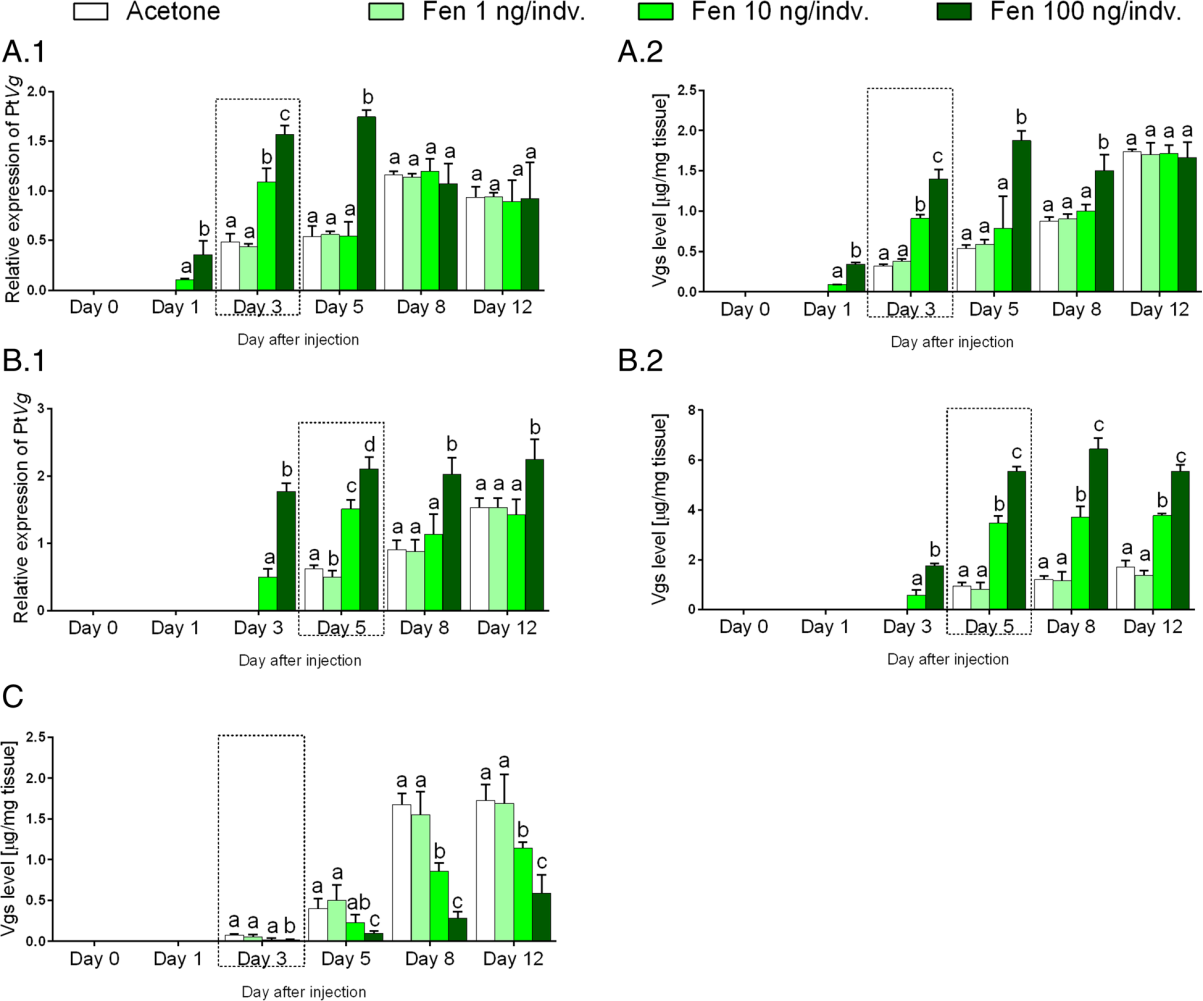
Level of vitellogenin (at transcript and protein levels) in response to fenoxycarb administration. The profile of the vitellogenins [mean ± SD] of the *P. tepidariorum* females that were treated with acetone and 1, 10 and 100 ng of fenoxycarb per individual. Vg results in the midgut glands at transcript (**a**.1) and protein levels (**a**.2), the ovaries at transcript (**b**.1) and protein levels (**b**.2) and the hemolymph at the protein level (**c**). The dashed line box shows the physiological time of vitellogenesis. Lowercase letters indicate significant differences between experimental groups at the same time point (Tukey’s multiple comparisons test, *p* ≤ 0.05)

Changes in the Pt*Vg*4 expression level and Vg concentration in all tested tissues compared to the control group after the fenoxycarb application in two highest doses (10 ng and 100 ng/indv) were observed (Fig. 4). Pt*Vg*4 expression and Vg presence in the midgut glands (respectively, Fig 4a1 and a2) and ovaries (respectively, Fig 4 b1 and B2) but not in the hemolymph in groups treated with fenoxycarb were reported 2 days earlier than in the control group. Similar results were obtained for females treated with 20E (compare with Fig. 3a and b).

The greatest difference between the level of tested parameters between the experimental group and the control group was demonstrated in the group treated with the most effective dose of fenoxycarb (100 ng/indv). The 3.5-time higher level of tested parameters was observed for the midgut glands on day 5 of the experiment (Fig 4a1 and a2). Similarly, the greatest increase for the ovaries compared to the control group was demonstrated for the Vg expression level on day 5 of the experiment (3.5-time higher level; Fig. 4b1) and the Vg concentration on day 8 (5.5-time; Fig. 4b2).

It seems that fenoxycarb acts differently on the midgut glands than on the ovaries activity. Changes in the Pt*Vg*4 expression level and Vg concentration in the midgut glands compare with the control group were only observed until respectively the day 5 and 8 of the experiment (Fig. 4a). On the other hand, the effect of fenoxycarb at transcript and protein levels in the ovaries was visible until the end of the duration of the experiment (Fig. 4b).

It seems that fenoxycarb acts on the Vg at the protein level in the hemolymph in another way than in the midgut glands and the ovaries. Administration of the fenoxycarb caused a dramatic reduction of the Vg concentration in the hemolymph. Even 6-time reduction of the Vg level after application of the fenoxycarb at a dose of 100 ng/indv was observed on day 8 of the experiment (Fig. 4c).

### Effect of precocene I on Pt*Vg*4 expression and vg concentration

**Fig. 5:**
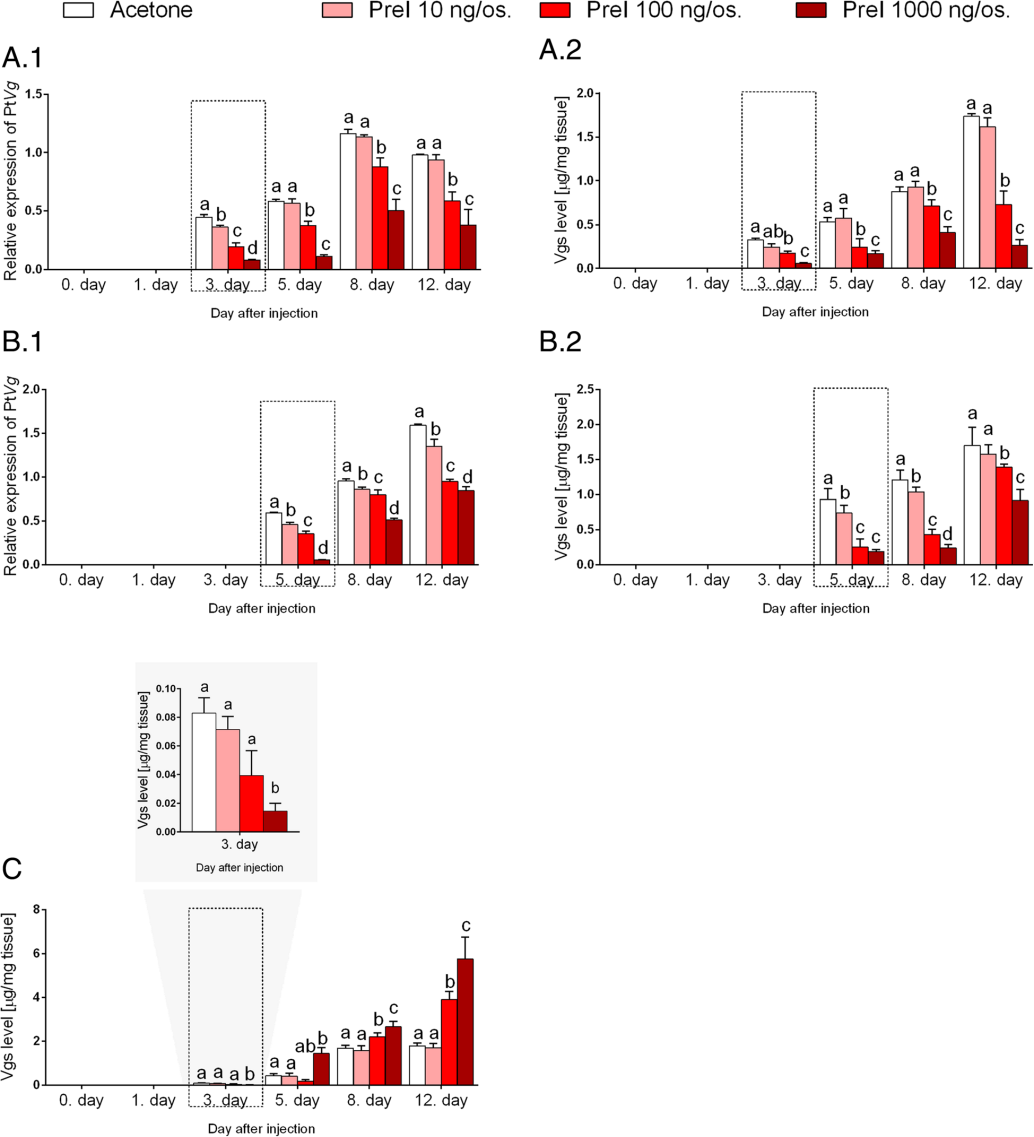
Level of vitellogenins (at transcript and protein levels) in response to precocene I administration. The profile of the vitellogenins [mean ± SD] in the *P. tepidariorum* females that were treated with acetone and 10, 100 and 1000 ng of precocene I per individual. Vg results in the midgut glands at transcript (**a**.1) and protein levels (**a**.2), the ovaries at transcript (**b**.1) and protein levels (**b**.2) and the hemolymph at the protein level (**c**). The dashed line box shows the physiological time of vitellogenesis. Lowercase letters indicate significant differences between experimental groups at the same time point (Tukey’s multiple comparisons test, *p* ≤ 0.05)

The application of the precocene I caused changes in the expression profile of the Pt*Vg*4 and in the Vg level in all tested tissues, compared with the control group (Fig. 5). The earlier expression of the Vg at transcript and protein levels than in control was not observed in experimental groups, which was the opposite of bioassay results with other compounds (compare with Figs. 3 and 4).

The decrease in the Pt*Vg*4 expression level and Vg concentration in the midgut glands and ovaries was caused by the precocene I application. The degree of the Vg decrease was dependent on the dose that was used: the higher dose – the lower level of the Pt*Vg*4 transcript and Vg concentration. The highest dose of precocene I (1000 ng/indv) caused the highest dramatic decrease in the Pt*Vg*4 expression level (5.5-time in the midgut glands on the day 3 of the experiment and 10-time in the ovaries on the day 5 of the experiment) (respectively, Fig 5a1 and b1) and in the Vg concentration (6.5-time in the midgut glands on the day 12 of the experiment and in the ovaries on the day 5 of the experiment) (respectively, Fig 5a2 and b2) in both tested tissues.

A different effect of precocene on the Vg level was demonstrated in the hemolymph. In general, the increase in the Vg concentration was observed after the precocene I application compare with the control group (Fig. 5c). The most effective dose of precocene (1000 ng/indv) caused a 3.25-time increase in the Vg level in the hemolymph compare with control group. On the other hand, there was a decrease in the Vg concentration on day 3 after compound application at a dose of 1000 ng/indv.

Analysis of all results obtained in bioassays revealed strong and very strong correlations between the Pt*Vg*4 expression level and Vg concentration in tissues and the time of tissue collection in all of the experimental groups treated with 20E, fenoxycarb and precocene I (Table 1).

**Table 1:** Correlation between the Vg level in experimental groups and the time point of tissue collection

Tissue	Compound	Dose of the compound applied [per indv]	Pt*Vg*4 level		Vgs concentration	
			*r*	*p*	*r*	*p*
Midgut glands	20-hydroxyecdysone	10 ng			0.867	0.001
		100 ng				
		200 ng			0.976	0.001
Ovaries		10 ng	0.98	0.001	0.825	0.031
		100 ng	0.99	0.000	0.986	0.002
		200 ng	0.99	0.000	0.966	0.002
Hemolymph		10 ng	x	x	0.845	0.032
		100 ng	x	x	0.986	0.002
		200 ng	x	x		
Midgut glands	Fenoxycarb	1 ng	0.91	0.013	0.989	0.001
		10 ng			0.945	0.005
		100 ng			0.815	0.005
Ovaries		1 ng	0.98	0.001	0.945	0.005
		10 ng	0.84	0.03	0.901	0.024
		100 ng	0.82	0.002	0.862	0.027
Hemolymph		1 ng	x	x	0.951	0.004
		10 ng	x	x	0.963	0.013
		100 ng	x	x	0.843	0.012
Midgut glands	Precocene I	10 ng	0.908	0.012	0.993	0.000
		100 ng			0.956	0.003
		1000 ng			0.905	0.000
Ovaries		10 ng	0.98	0.002	0.972	0.001
		100 ng	0.961	0.002	0.927	0.008
		1000 ng	0.951	0.002	0.913	0.011
Hemolymph		10 ng	x	x	0.946	0.005
		100 ng	x	x	0.938	0.006
		1000 ng	x	x	0.961	0.002

The correlation between Pt*Vg*4 expression level and Vg concentration in tissues (midgut glands, ovaries and hemolymph) and the time of tissue collection in various experimental groups that were treated with 20E, fenoxycarb and precocene I at different doses

The scale of the assessment of the correlation the *r* values: 0.2–0.4 – weak, 0.4–07 – average, 0.7–0.9 – strong, 0.9–1 – very strong

x – no analysis,

empty fields – no statistically significant correlation

## Discussion

### Vitellogenesis – Site and time of the vg synthesis

In our research, we revealed in silico the presence of two genes encoding putative vitellogenin in the genome of *P. tepidariorum* spiders (Pt*Vg*4 and Pt*Vg*6). However, Pt*Vg*4 was only confirmed to encode Vg. Moreover, data from NCBI Protein database [43] suggests the presence of three genes encoding Vgs in *P. tepidariorum* spiders (vitellogenin-4-like isoform X1 of *P. tepidariorum*, accession no. XP_015930209 and vitellogenin-6 *P. tepidariorum*, accession no. XP_015930207, vitellogenin-4-like isoform X2 of *P. tepidariorum*, accession no. XP_015930210). Therefore, further studies on the existence of other *Vg* genes in the spider genome are required to gain a better understanding of the vitellogenesis in these spiders. Especially because three genes encoding the Vg in spider *P. pseudoannulata* has been reported by Guo et al. [26].

We also determined three subunits of the Vg at the protein level (250 kDa, 47 kDa and 30 kDa) in the midgut glands, hemolymph and ovaries of female spiders. In arthropods, each Vg subunit has a cleavage signal motif that is recognized by endoproteases. The presence of a few Vg subunits results from a modification of the number of domains with cleavage signal motifs and their localization in the primary product of the Vg gene [2, 45, 46]. Guo et al. [26] revealed many possible cleavage motifs in the sequence of the Vg genes in spider *P. pseudoannulata* (for *Vg*1 – seven motifs, *Vg*2 – six and for *Vg*3 – five motifs). These results may indicate a greater number of the Vg subunits than the number of genes encoding the Vg in spiders. For tick, the lack of dependence between the number of Vg genes and the number of Vg subunits is common. For example, Thompson et al. [28] reported 7 subunits of Vgs in the tick *D. variabilis* despite only two genes encoding Vgs in this species of tick are observed.

Pourié and Trabalon [35] showed the presence of the putative 47 kDa-Vg protein in the female hemolymph of the *Eratigena atrica* spider. This is in accordance with our results and may also indicate the presence of a 47 kDa subunit protein in all spiders. This hypothesis requires further research based on interspecies comparisons.

Analysis of the expression profile of the Vg at transcript and protein levels made it possible to draw conclusions that until now were unknown to science. The presence of the Pt*Vg*4 transcript and the Vg in the midgut glands indicate that this tissue is the main site for Vg synthesis in *P. tepidariorum* females during the tested period of the development (between the penultimate nymphal stage and the day of the first oviposition). The midgut is the place of the Vg synthesis in a number of tick species. However, the Vg is also produced by the tick fat body that does not exist in spiders [6, 7, 28–30].

The last nymphal stage (day 38 after leaving a cocoon) is the time at which vitellogenesis began in the midgut glands of *P. tepidariorum*. The results that were obtained contradict the studies of Hilbrant et al. [27] and Guo et al. [26]. Hilbrant et al. [27] claimed that vitellogenesis begins on the day when sexual maturity is reached (day 40 after leaving a cocoon) in *P. tepidariorum* females. In our opinion, the methods that were used in our study are more precise than the behavioral observations that were used by Hilbrant et al. [27]. The time used by Hilbrant et al. [27] is the moment when (as our results showed) the Pt*Vg*4 transcript appeared in the ovaries and when the size of the female abdomen increased. However, it should be noted that the Vg is synthesized in the midgut glands of *P. tepidariorum* (similar to the fat body of insects [31]) and, according to our results, this process begins in the last larval stage in this tissue. Whereas, Guo et al. [26] have observed the presence of the Vg transcript in the whole body extract of the sixth-instar spiderling, sub-adult and 2 days post-maturation female of the spider *P. pseudoannulata*. The authors do not specify how many instar stages this species of spider have in their breeding. In addition, there are no data what does mean ‘the sixth-instar spiderling’. Does sixth-instar correspond to the penultimate nymphal stage of the *P. tepidariorum*? If yes, it can be indicated that the vitellogenesis begins earlier in the species of spider studied by Guo et al. [26].

The presence of the Pt*Vg*4 transcript in the ovaries shows that the Vg is also synthesized by this tissue, but only in spiders after mating (day 40 after leaving a cocoon). This indicates that the mating process plays a crucial role in triggering vitellogenesis in the ovaries. Furthermore, it also seems that the nymphal stage and virgin females of *P. tepidariorum* (until the day 40) may maintain ovaries in the pre-vitellogenic phase while mated females may contain ovaries in the vitellogenesis. The crucial role of the mating in the spider ovarian and oocyte development is noted by Trabalon et al. [24, 25]. It should be emphasized that the ovaries function in this process gradually increases during the development of the spider and expression of the Pt*Vg*4 exceeded that in the midgut glands the day of the first oviposition (day 47 after leaving a cocoon). Ovaries may gradually assume the main role of Vg synthesis. The role of the ovaries in the Vgs synthesis is commonly known for some species of insects (e.g. for *Drosophila melanogaster*) [47]. On the other hand, studies on ticks clearly show that the midgut and fat body are the only sources of the Vgs in this group of Arachnida [7, 29, 48]. However, the expression of the Vg gene in ovaries of *H. longicornis* was observed by Boldbaatar et al. [6]. Furthermore, de Oliveira et al. [4, 5] studies of the ovaries morphology indicate the role of the ovaries and the pedicel cells of *Rhipicephalus sanguineus* and *Amblyomma cajennense* connected with oocytes in the Vg synthesis (respectively, autosynthesis and heterosynthesis of the Vg). Thus it seems that our results should be enriched with morphological studies of the *P. tepidariorum* ovaries because it is necessary to check the role of the pedicel cells in the Vg synthesis.

Similar to insects and ticks [46, 49], the spider hemolymph is probably responsible for the transport of Vg between the midgut glands and ovaries. It also seems that the Vg secretion process from the midgut gland begins in the last nymphal instar (day 38 after leaving a cocoon) because that is the day of the Vg appearance in the hemolymph. It is possible that the secretion coincides with the beginning of the Vg heterosynthesis in the midgut glands, which proves that the Vg that is produced in this tissue is not stored, but rather are secreted into the hemolymph. An intensification of the Vg transport occurred between days 40 (mating) and 47 (day of the first oviposition) after leaving a cocoon.

Fluctuations in the Pt*Vg*4 transcript level and the Vg concentration correspond with changes in the body of the female spiders that are necessary for successful reproduction. The significant increase in the tested parameters from the day that sexual maturity was reached and mating began indicates an important role for mating in the regulation of vitellogenesis. Similar results have been reported for *P. pseudoannulata* by Guo et al. [26] but in the whole body extract and not in the ovaries. The expression level of two Vg genes was significantly expressed on day 1 after mating in this species of the spider. Furthermore, the increase of the Pt*Vg*4 transcript level and the Vg concentration show a possible important function of the Vgs for the developing spider embryos, which is similar to insects and ticks [1, 46, 50].

In conclusion, both exogenous and endogenous synthesis (autosynthesis) of Vg take place in *P. tepidariorum* females. Like other arthropods, exogenous vitellogenesis occurs in the most metabolically active tissue (in spider midgut glands) while autosynthesis occurs in the ovaries [51, 52]. It is possible that the tick Vg is synthesized in four places (exogenously in the midgut, fat body and in the pedicel cells, and endogenously – in the oocytes) [4, 5]. Therefore, the morphological studied of the *P. tepidariorum* ovaries are needed to determine the role of the pedicel cells in the Vg synthesis.

**Fig. 6:**
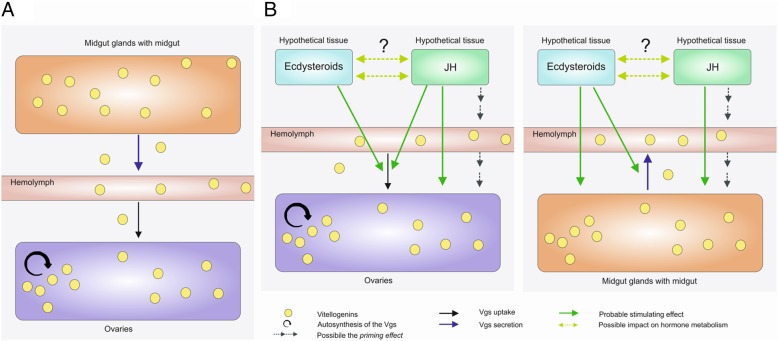
Hypothetical model of vitellogenesis and its hormonal control in *P. tepidariorum* spiders. **a** Organs and tissues responsible for the synthesis and transport of the Vg with a crucial role the midgut glands and ovaries. **b** The probable role of hormones in the vitellogenesis. Heterosynthesis of the Vg in the midgut glands is probably under control of the juvenile hormone and ecdysteroids (indicated by green arrows). On the other hand, the juvenile hormone may be only one hormone that is responsible for the control of the Vg autosynthesis in the ovaries. The Vg secretion from the midgut glands into the hemolymph (indicated by a blue arrow) is probably controlled by ecdysteroids. The Vg uptake by oocytes (indicated by a black arrow) may be regulated by the juvenile hormone and ecdysteroids. It should be remembered about the possible *priming effect* (indicated by dashed arrows) induced by the juvenile hormone. As literature data indicate the one hormone can affect the metabolism of the other hormone. Thus this possible action of hormones in *P. tepidariorum* is indicated by an arrow with two caves

The key findings of the study about the vitellogenesis *in P. tepidariorum* are presented in Fig. 6a.

### Hormonal control of vitellogenesis

Knowledge about the hormonal regulation of vitellogenesis in spiders is based on scarce and conflicting reports. A few literature studies have indicated that ecdysteroids are responsible for regulating this process. Trabalon et al. [24] revealed that the transition from pre-vitellogenesis to vitellogenesis in *Coelotes terrestris* and *E. domestica* spiders is correlated with an increase in ecdysteroid (ecdysone and 20E). Moreover, Trabalon et al. [25] and Pourié and Trabalon [35] observed that the administration of exogenous 20E affects the induction and regulation of vitellogenesis in these species of spiders. On the other hand, Stubbendieck et al. [36] indicated a potential lack of any role of ecdysteroids in initiating the process of vitellogenesis at least in the spiders of the *Schizocosa* genus. The role of juvenile hormones and their precursors in the spider vitellogenesis is also as yet unknown.

Results of our bioassays indicate that vitellogenesis in *P. tepidariorum* may be regulated by ecdysteroids and the juvenile hormone. Comparative analysis of the Pt*Vg*4 expression and the Vg level after hormonal treatment show that these hormones may cooperate in the vitellogenesis regulating. Thus, our results are the basis for proposing a hypothetical model of the hormonal regulation of vitellogenesis in *P. tepidariorum* females. However, the model should be confirmed in further studies.

The discussion of the bioassay results is based on the most effective doses of used compounds, as well as the significant time points at which the tissue was collected (days that had elapsed from the injection/topical application, which are closely related to the spider age). These doses were determined based on their impact on the Pt*Vg*4 expression level and Vg concentration and the duration of these effects compared to the control group. According to our results, the most effective doses were: 20E – 100 ng/indv, fenoxycarb – 100 ng/indv and precocene I – 1000 ng/indv. The effect of these compounds doses was observed 2 days earlier than in the physiological condition of the tissue (apart from hemolymph). Moreover, all of the tested compounds at the most effective doses had a long-term effect on the Pt*Vg*4 expression level and Vg concentration until day 12 of the experiment in all tissues. However, the juvenile hormone analog – fenoxycarb was only effective up to day 8.

### Synthesis of the vg

The 20E administration was connected with the significant increase of the Pt*Vg*4 expression and the Vg concentration in the midgut glands and ovaries. The Vg appeared 2 days earlier in both organs in the experimental group than in the control group. Therefore, It seems that 20E is necessary for the vitellogenesis in *P. tepidariorum*. However, 20E administration was associated with a significantly higher increase of the Pt*Vg*4 expression level and Vg concentration in the midgut glands than in the ovaries. It may indicate the marked role of the 20E in the vitellogenesis in the midgut glands (heterosynthesis) and minor role of the 20E in the ovaries. Results obtaining in groups treated with the fenoxycarb confirm this hypothesis. The fenoxycarb treatment was connected with the 2 days earlier appearance of the Vg at the transcript and protein levels (similar to groups treated with 20E) in the midgut glands. However, a greater increase in the protein level was demonstrated for the 20E treated group (e.g. 1.6-fold more the Vg transcript on day 3 than in the fenoxycarb group). The dominant role of the 20E in the vitellogenesis induction is observed in the tick fat body (e.g. in *Ornithodoros moubata* [40, 42], *D. variabilis* [15, 20], *A. hebraeum* [41]).

On the other hand, the juvenile hormone may be necessary for the vitellogenesis in the midgut glands. The precocene I treatment was connected with a strong, more than 5-fold decrease in the Pt*Vg*4 expression level and Vg concentration in the physiological conditions at the time that vitellogenesis began (in the control group). The specific role of the juvenile hormone in the heterosynthesis of the Vg is reported for insects from orders Hymenoptera (except for social insects) and Diptera (including *D. melanogaster* and *A. aegypti*) [11]. In these insects, the juvenile hormone is directly responsible for the induction of the Vg synthesis in ovaries. However, the second indirectly role is based on the stimulation of the 20E synthesis in ovaries. Then, the 20E induces the Vg synthesis in the fat body. The juvenile hormone is also responsible for *the priming effect*. *The priming effect* enables a cell to effectively respond to the juvenile hormone or ecdysteroids [13, 53].

It seems that the juvenile hormone is also necessary for the vitellogenesis in *P. tepidariorum*. However, although the fenoxycarb induces the appearance of the Vg at the transcript and protein levels 2 days earlier than in the control group in both organs, the juvenile hormone may play a marked role in the ovaries and not in the midgut glands. The fenoxycarb administration and not 20E was associated with the highest increase in the Pt*Vg*4 expression level and Vg concentration in the ovaries. For instance, the Pt*Vg*4 expression level was 3.3-fold more in the fenoxycarb-group treated than in the 20E-group in day 3 in this organ. Furthermore, the effect of the fenoxycarb treatment for the Vg level remained constant until the end of the experiment.

The dominant role of the juvenile hormone in the inducing the Vg synthesis in the ovaries was demonstrated e.g. in *D. melanogaster* [11]. The juvenile hormone is responsible for the stimulation of the Vgs autosynthesis in the ovaries [16]. In addition, it also enables cells to efficiently respond to the juvenile hormone and to effectively produce Vgs (*the priming effect*) [13]. On the other hand, data about ticks indicate that the juvenile hormones (if they exist) do not play a role in the stimulation of the Vg synthesis in the ovaries [39].

It is possible that the autosynthesis of the Vg may be not only dependent the juvenile hormone but other factors. The vitellogenesis occurred in the ovaries even when the precocene was administered. As described above, the 20E may be the additional factor, because the Pt*Vg*4 expression and the Vg concentration were connected with the 20E administration. As the literature data based on *D. melanogaster* shows, 20E can affect the juvenile hormones metabolism by inhibiting their degradation and increasing their titer. Thus, 20E can indirectly stimulate the Vgs autosynthesis [19].

### Secretion, transport and deposition of the vg

Deposition of the Vg in the developing oocytes is one of the most important processes of vitellogenesis [18]. The Vg concentration in the hemolymph indicates the efficiency of heterosynthesis, secretion of Vg into the hemolymph and their transport and deposition in the ovaries [10]. A high concentration of Vg in the spider hemolymph may indicate a strong induction of the secretion of these proteins from the midgut glands, but may also indicate the inefficient Vg transport, as well as a low level of the intensity of the Vg deposition in the ovaries.

The 20E treatment was connected with a higher Vg concentration in the hemolymph. Similar results have been observed by Pourié and Trabalon [35] in *E. atrica*. However, the Vg fluctuation pattern in the hemolymph was similar to that in the midgut glands in the same experimental group. These results raise the question about the possible role of the 20E in the Vg secretion into the hemolymph. Does 20E play a role in the stimulation of the Vg secretion into hemolymph or rather the Vg synthesis in the midgut glands?

The 20E treatment was also connected with a simultaneous Vg level increase in the ovaries as well as with a decrease in its concentration in the midgut glands and hemolymph. This may indicate the role of 20E in inducing of the Vg deposition in the ovaries. However, the data about ticks indicate that ecdysteroids are not responsible for depositing them in the ovaries [41] but rather for inducing the Vg secretion into the hemolymph [54]. On the other hand, the role of the 20E in the Vg deposition in oocytes was confirmed in e.g. Diptera [11, 16].

The fenoxycarb application was related with a significant decrease in the Vg level in the hemolymph, although at the same time the Vg level in the midgut glands and in the ovaries increased. It can be a result of a stimulating role of the juvenile hormone in the Vg synthesis in the midgut glands and ovaries, as suggested above. However, the precocene I treatment was connected with a decrease in the Vg level in the midgut glands, the accumulation of these proteins in the hemolymph and a decrease in the Vg concentration in the ovaries. Therefore, these results may indicate the major role of the juvenile hormone in the Vg deposition.

Similar results were obtained for insects (*D. melanogaster*, *Locusta migratoria*) as well as for ticks (e.g. *O. parkeri*), thus indicating that the juvenile hormone is necessary for the induction of Vg secretion and deposition in ovaries in this species of arthropod [9, 10, 13, 19]. On the other hand, it should be noted that Lunke and Kaufman [55] have not observed the role of the juvenile hormone in the ovaries Vg deposition of *A. hebraeum* tick.

### Hypothetical model of the hormonal regulation of vitellogenesis

Presented results are the first wide-ranging report about the hormonal control of vitellogenesis in spiders. Obtained results not only enabled us to propose a number of hypotheses, but also to prepare a hypothetical model for the hormonal regulation of vitellogenesis in *P. tepidariorum* females (Fig. 6b).

It seems that both ecdysteroids and juvenile hormone can be able to regulate vitellogenesis. A similar model was demonstrated in insects from the orders Hymenoptera (except for social insects) and Diptera (including *D. melanogaster* and *A. aegypti*) [11]. It should be noted that the dissimilar model of the vitellogenesis regulation was observed in ticks. In this group of arthropods, the crucial role of the juvenile hormone in the vitellogenesis is confirmed [20, 29, 41, 54, 56].

## Conclusions

Two genes encoding Vg (Pt*Vg*4 and Pt*Vg*6) in the genome of the spider *P. tepidariorum* are revealed. One gene Pt*Vg*4 and three subunits of Vg (250 kDa, 47 kDa and 30 kDa) are expressed in the midgut glands, ovaries and hemolymph. Heterosynthesis of the Vg in the midgut glands and autosynthesis in the ovaries were observed. Vitellogenesis begins in the last nymphal stage in the midgut glands (heterosynthesis). However, after sexual maturity is reached, Vg is also synthesized in the ovaries (autosynthesis). In our opinion, the obtained results can be consistent with the data that was obtained by Guo et al. [26] and are contradict the data from Hilbrant et al. [27] studies. In addition, they provide answers to the questions that were included in the papers: Sawadro et al. [32] and Pourié and Trabalon [35].

Results of our bioassay tests indicated that both ecdysteroids and juvenile hormone can be able to regulate vitellogenesis in *P. tepidariorum*. Obtained results not only enabled us to propose a number of hypotheses, but also to prepare a hypothetical model for the hormonal regulation of vitellogenesis in *P. tepidariorum* females. Presented results are the first wide-ranging report about the hormonal regulation of vitellogenesis in spiders.

Results filled the gaps in knowledge about spider vitellogenesis and hormonal regulation of this process. It should be noted that they will be an additional opinion in the discussion about the presence of juvenile hormones in ticks and their role in the vitellogenesis.

## Methods

### Spiders

Both sexes of the *Parasteatoda tepidariorum* spider C. L. Koch, 1841 (Araneae, Theridiidae) from the laboratory-bred strains of the Department of Animal Physiology and Ecotoxicology from University of Silesia were selected as the model organisms. Each spider has been bred separately in a transparent plastic tube. In order to ensure subsequent generations of the breeding line, the sexually mature individuals (females on day 40 after leaving a cocoon and males on day 30) were paired and the mating was being observed. Males were left for few hours to allow possible next mating. After this time, the males were isolated. The cocoons were carried to a new container, and the nymphs were transferred to separate plastic tubes. The animals were bred at 25 ± 1 °C at a 70% relative humidity under a L: 16 h, D: 8 h photoperiodic cycle. They were fed lab *Drosophila melanogaster* or *D. hydei* and watered regularly (three times a week).

A well-fed adult female lays ten cocoons (on average) and each sac contains up to 500 eggs. Duration of embryonic development is 8 days at 25 °C. Postembryo remains in the cocoon about 2 days. Next, the first moult is observed and gives the first nymphal stage that leaves the cocoon. The time from leaving the cocoon to reaching maturation takes approximately 30 days for males (four to six moults) and 40 days for females (with five to seven moults) [27, 57]. Under the strict regime of breeding condition, the majority of females has six moults and males – five moults (personal report). Two last nymphal stages can be visually distinguished on the basis of morphological characteristics, e.g. epigyne and palps, the size of the abdomen, the ratio of the size of the cephalothorax to the abdomen, the colour of the body [27, 57].

### *Vg* gene expression

#### In silico search

Vitellogenin amino acid sequences that were used in the search for the *Vg* gene (or genes) in the *P. tepidariorum* genome were from following species (accession number in parenthesis). Insects – Blattodea: *Blattella germanica* (CAA06379), Hymenoptera: *Apis mellifera* (NP_001011578 XP_392349), Diptera: *Drosophila melanogaster* (AAF46547), Lepidoptera: *Bombyx mori* (NP_001037309 NP_001037310), *Spodoptera exigua* (AOH73254); Arachnida – Ixodida: *Dermacentor variabilis* (AAW78557), *Ornithodoros moubata* (BAH02666), *Amblyomma hebraeum* (AGQ57040), *Rhipicephalus appendiculatus* (JAP78589), Trombidiformes: *Tetranychus cinnabarinus* (ANS13820); Malacostraca – Decapoda: *Macrobrachium rosenbergii* (BAB69831), *Homarus americanus* (ABO09863) were collected from the NCBI protein database [43]. These sequences were used as queries in order to identify the putative gene homologs of the *P. tepidariorum Vg* gene (genes) via a tblastn search [58] in the Transcriptome Shotgun Assembly database, which is based on the sequence homology criterion that was proposed by Pearson [44]. The transcript sequences that were selected in this way were transcribed into protein sequences using the Geneious® (ver. 9.1.2) software. These sequences were used to confirm the identity of the putative *Vg* gene in *P. tepidariorum* with *Vg* gene sequences of other arthropods. For this purpose, the Geneious®, Clustal Omega and InterProScan [59] software and algorithms were used. Starters for the further analysis of the section of putative genes encoding the Vgs in *P. tepidariorum* were designed using the Geneious® software.

### Vg gene expression

The sex-specific expression of the putative Vg gene was studied by the PCR reaction analysis. For this purpose, the total RNA from the whole body extract of both sexes of *P. tepidariorum* was isolated using TRIzol® Reagent (Invitrogen™, Carlsbad, CA, USA) following the manufacturer’s protocol. One adult female and two males on the first day after mating (for female – day 41 after leaving a cocoon and for male – day 31 after leaving a cocoon) per sample were used. After TURBO DNAse (Ambion, Austin, TX, USA) treatment, 1 μg of RNA was used for cDNA synthesis using the Reverse Transcription System (Promega, Madison, WI, USA) and random primers. The PCR reaction was performed using the PPP Master Mix (Top-Bio, Vestec, Czech Republic) and the starters from the in silico analysis (F: 5′-CAGTGCGATGTACGGATAT-3′, R: 5′-TGTCTTCTGCTGTGAGTTG-3′) were used. Reactions were performed in 25 μl total volume with 4 ng cDNA, 2.5 U Taq Purple DNA polymerase and 20 mM each of the primers under the following thermal conditions: 95 °C for 5 min, 36 cycles at 95 °C for 30 s, 55 °C for 35 s, 72 °C for 45 s and termination at 72 °C for 10 min. Each sample had 20 biological replicates (*n* = 20) and three technical repetitions. For this part of the study, the whole body extract of 40 male and 20 female of spiders were used. Further analyses were based only on females.

Obtained results were the starting point for the next step of the experiment. Tissues of the female spiders where the Pt*Vg*4 gene is expressed was determined using the PCR method. cDNA templates and the PCR protocol were similar to those described above. The nervous system (supraesophageal ganglia and subesophageal ganglia) with the neuroendocrine system (NS + NS), ovaries (OV), midgut glands (MG), hindgut (HG) and integument (INT) of the female spiders on the day of the first oviposition (day 47) were used. Spiders were anesthetized 5 min before dissection on ice. The tissues and organs used for the Vg expression studies were dissected on ice in a sterile condition with sterile phosphate-buffered saline (PBS, 137 mM NaCl, 10 mM phosphate buffer (K_2_HPO_4_, KH_2_PO_4_), 2.7 mM KCl, pH 7.4). All tissues and organs were immediately frozen in liquid nitrogen and stored at − 70 °C in the sterile Eppendorf tubes until use.

The following tissues and organs: the hindgut, nervous system with the neuroendocrine system and integument were chosen as a probable negative control (lack of data about the Vg expression in these tissues in arthropods [60]). The midgut glands with midgut and ovaries was were tested as a probable place of the vitellogenesis in *P. tepidariorum* (based on data about ticks – (e.g. [6, 7]).

The number of animals used per one sample from various tissues is as follow for each sample from: the nervous system with neuroendocrine system and hindgut – 11 spiders, ovaries, midgut glands, and integument – 5 spiders. Each sample had six biological replicates (*n* = 6) and three technical repetitions. For this part of the study, the 66 female spiders were used. The midgut glands and ovaries were used for further analysis.

The stage-specific Pt*Vg*4 expression was studied in the midgut glands and ovaries using quantitative real-time PCR (qPCR) analysis. The female spiders in the crucial ages were used. Age of spiders was selected based on behavioral and morphological observations of their life cycle and counted from leaving the cocoon according to Miyashita [57] and they were: the penultimate nymphal stage (day 35 after leaving a cocoon), last nymphal stage (day 38), the day that sexual maturity was reached (day 40), 3 days after mating (day 43) and the day the day of the first oviposition (day 47). Tissues were collected in the same way as for the tissue-specific expression of the putative Vg gene (described above). Tissues from 260 female spiders were used in this stage of the experiment. The number of animals that were used per one sample from tested tissues and different ages are presented in Table 2. Each sample had six biological replicates (*n* = 6) and four technical replications.

**Table 2:** Number of female spiders per sample that were used to determine the Pt*Vg*4 expression

Stage of tested development	Number of spiders per sample	
	Midgut glands	Ovaries
Penultimate nymphal stage	10	20
Last nymphal stage	5	10
Day of sexual maturity was reached	5	5
Three days after mating	5	5
Day of the first oviposition	3	3

The number of *P. tepidariorum* females that were used to prepare one sample from the midgut glands and ovaries to determine any fluctuations in the *Vg* expression at various ages

Real-time fluorescence data of reactions containing SYBR Green I were performed using a LightCycler 480 (Roche, Basel, Switzerland) using the SYBR™ Select Master Mix (Applied Biosystems™, Foster City, CA, USA). Each cDNA sample was analyzed in a 15 μl reaction with 100 ng cDNA (obtained from tissues in the same way as the template described above) and 200 nM each of the primers in 96-well white plates (Roche, Basel, Switzerland) under the following thermal conditions: 95 °C for 3 min and 40 cycles; 95 °C for 15 s, 57 °C for 20 s, 72 °C for 45 s. *rp49* was used as the reference gene (F: 5′-GCACTAAGACCATTAGTTAGC-3′, R: 5′-CGGAGAGATTTCAGCACATAC-3′) after the preliminary tests of the most popular housekeeping genes in arthropods (rp18, α-actin, β-actin, not shown). Quantification of the Pt*Vg*4 expression was calculated according to the standard curve methods [61]. Standard curves for the Pt*Vg*4 and Pt*rp49* genes of five dilution series (between 10^10^ to 10^6^ copies of the DNA molecules) were constructed from purified cDNA (using a QIAquick PCR Purification Kit and following the manufacturer’s protocol) that were obtained from a previous PCR using the same primer set. A melting curve analysis was performed in order to ensure the homogeneity of the product. cDNA templates from the whole body extract of the *P. tepidariorum* males on the first day after mating were used as the negative control and the cDNA from *P. tepidariorum* eggs was used as the positive control.

The Pt*Vg*4 level linearity in various tissues during the tested part of the spider development was measured by determining the *R*^*2*^ value. The Pearson correlation for determining the relationship between the changes in Pt*Vg*4 level and the age of spiders was used. The Pt*Vg*4 expression between spiders in various ages was compared using Tukey’s multiple comparisons test.

### Vg proteins profile

In order to determine the Vg presence and concentration in various tissues in *P. tepidariorum* and to confirm the results from the Pt*Vg*4 gene expression analysis, studies consisting of three steps at the protein level were performed.

In the first stage, the protein profile in various tissues was determined using Sodium dodecyl sulfate polyacrylamide gel electrophoresis (SDS-PAGE) under denaturing conditions according to Laemmli [62]. The aim of this stage was to identify the subunit/subunits of putative Vg of *P. tepidariorum*. Ovaries from females in last nymphal stage (day 38), the day that sexual maturity was reached (day 40) and 3 days after mating (day 43 after leaving a cocoon) were used. In this stage of the experiment, the ovaries from 42 female spiders were used. The number of ovaries for one sample depended on the age of females and is presented in Table 3. Each sample had six biological replicates (*n* = 6). Moreover, all two-day-old eggs from 25 cocoons (egg mass of each cocoon was one sample, *n* = 25) were used as the positive control and the whole body extract of a male after mating (*n* = 25) was used as the negative control.

**Table 3:** Number of female spiders that were used to study the Vg at the protein level

Stage of tested development	Number of female spiders per sample			
	SDS-PAGE	ELISA		
	Ovaries	Midgut glands	Ovaries	Hemolymph
Penultimate nymphal stage	–	5	6	10
Last nymphal stage	3	3	3	6
Day that sexual maturity was reached	2	2	2	4
Three days after mating	2	1	2	3
Day of the first oviposition	–	1	1	3

Number of *P. tepidariorum* females that were used to prepare one sample from the midgut glands, ovaries and hemolymph to determine the Vg at the protein level at various ages

Ovaries, eggs and the whole body extract of a male after mating were dissected on ice after 5 min anesthesia of spider on ice. Then organs were homogenized in 200 μl of phosphate-buffered saline (PBS), pH 7.4. Samples were homogenized in 1.5 mL Eppendorf tubes using a hand-held homogenizer. All of the samples were centrifuged at 10,000 rpm for 10 min at 4 °C. Supernatants were stored at − 70 °C until the measurements were performed.

Total protein concentration in samples was determined according to Bradford [63] using bovine serum albumin (BSA, protein content > 95%, Fluka) as the standard. Samples were diluted to the same protein concentration using a PBS buffer, pH 7.4 and a sample buffer (according to Laemmli [62]: 62.5 mM, Tris-HCl, pH 6.8, 2% SDS, 10% glycerol, 5% β-mercaptoethanol and 0.001% bromophenol blue). Then, they were denatured at 94 °C for 5 min.

SDS-PAGE was conducted using a vertical slab gel apparatus. Ten percent separating gels, which were run with the variable voltage set from 50 V to 130 V, were used. A total of 15 μg of protein was loaded into each well. The gels were prepared in duplicate.

The first gel was fixed in 50% methanol and 10% glacial acetic acid for 1 h with gentle shaking, stained with a solution of 0.1% (*w*/*v*) Coomassie Brilliant Blue G-250, dissolved in a mixture of methanol:glacial acetic acid:water (5:1:4) with gentle agitation for 30 min and destained in a mixture of methanol:water:acetic acid (4.5:1:4.5). Relative molecular mass of electrophoretically separated proteins was determined using a PageRuler Broad Range Unstained Protein Ladder (Thermo Scientific, Waltham, MA, USA). The bands were quantified using densitometric image analysis (Gelscan 2.0 program, Kucharczyk, Poland).

In the second stage, in order to confirm the presence of Vg subunits, Western blot of the second gels from the SDS-PAGE analysis was performed. Proteins from the second gels were transferred to a nitrocellulose membrane (Whatman® Protran®, Sigma-Aldrich, St. Louis, MO. USA), blocked with 5% BSA for 1 h in Tris-buffered saline (TBS – 150 mM NaCl, 50 mM Tris pH 7.4) and incubated with the primary antibody Mouse anti-medaka Vg (polyclonal antibody, dilution 1:800, Biosense Laboratories AS, Bergen, Norway) at 4 °C with gentle shaking overnight. Then, three-times washes of the membrane for 10 min with TTBS (TBS with Tween-20 at a final concentration 0.1%) were performed. Next, the membrane was incubated in the secondary antibody solution (goat anti-mouse peroxidase-conjugated, dilution 1:1000, CosmoBio Co. Ltd., Tokyo, Japan) at room temperature for 1.5 h. Visualization of the reaction product was performed using the colorimetric detection with horseradish peroxidase (Peroxidase product detection systems, SIGMAFAST™ OPD, Sigma-Aldrich, St. Louis, MO. USA). Selection of the primary antibody was preceded by preliminary tests of several commercially available antibodies (results not shown). Gels were analyzed using densitometry in ImageJ® software [64].

Two stages of the studies described above were repeated three times.

Vg concentration in various tissues of the female spiders was measured using ELISA. The midgut glands, ovaries, and hemolymph were used in this part of the research. Tissues were collected from females in various ages (determined by the number of days after leaving a cocoon): the penultimate nymphal stage (day 35 after leaving a cocoon), last nymphal stage (day 38), the day that sexual maturity was reached (day 40), three days after mating (day 43) and the day of the first oviposition (day 47). Amount of tissue for one sample depended on the age of the females and is presented in Table 3. In this stage of the experiment, tissues from 156 female spiders were used. Each sample had a minimum of six biological replicates (*n* = 6) and three technical replications.

Ovaries and the midgut glands were collected in the same way as for the SDS-PAGE. In addition, the hemolymph sampling was carried out by the cutting off legs at room temperature. Drops of liquid were gathered in automatic pipettes with tips, which had previously been immersed in an anticoagulant buffer (0.14 M NaCl, 0.1 M glucose, 30 mM trisodium citrate, 26 mM citric acid, 10 mM EDTA, pH 4.6 (2:1, *v*/v) [65]). Next, the hemolymph was transferred into 1.5 mL Eppendorf tubes. The volume of collected hemolymph depends on the stage of development of the spider and ranges from 2 to 10 μl of hemolymph per individual. The total volume of collected hemolymph for analyzing was 20 μl. The number of female spiders needed for the sample is included in Table 3. Then, supernatants were prepared from the midgut gland with midgut, ovaries, and hemolymph in the same way as for the procedure for tissues that were used for the SDS-PAGE.

ELISA was performed according to a protocol that is optimized for spiders [66]. The same set of antibodies was used for Western blot but different dilutions were used (for the primary antibody – 1:1000 and for the secondary antibody – 1:2000). Vg standard curve was performed based on the dilution series of the standard (Vgs of medaka, Biosense Laboratories AS, Bergen, Norway), which were diluted in PBS (pH 7.4). The measurement was performed using a Tecan Infinite M200 Microplate reader at 405 nm. Results were expressed as the Vg concentration in μg/mg the midgut glands or ovaries and in μg/ml for the hemolymph.

The linearity of the Vg levels relative to the subsequent tested part of development was measured by determining the *R*^*2*^ value. The Pearson correlation for determining the relationship between the changes in the Vg concentration and the age of spiders was used. Vg concentration between the different age groups of the spiders was compared using Tukey’s multiple comparisons test.

### Bioassays

In order to determine the possible effect of ecdysteroids and juvenile hormone on the vitellogenesis in *P. tepidariorum* females, bioassays were performed. For this purpose, three different compounds were selected: 20-hydroxyecdysone (Sigma Aldrich, St. Louis, MO, USA), a juvenile hormone analog – fenoxycarb (Ethyl (2-(4 phenoxyphenoxy)ethyl)carbamate; Fluka) (Fisher Scientific, Hampton, NH, USA) and an antijuvenoid – precocene I (7-Methoxy-2,2-dimethyl chromene; Sigma Aldrich, St. Louis, MO, USA). Fenoxycarb is a compound that acts as the juvenile hormone [67] and precocene I cause the physiological effects of this hormone [68]. Compounds were dissolved in a Ringer solution (86 mM NaCl, 5.4 mM KCl, 3 mM CaCl_2_ x 2H_2_O) (for 20-hydroxyecdysone) or in acetone (in the case of fenoxycarb and precocene I; Sigma Aldrich, St. Louis, MO, USA) in order to yield 1 mg/μl stock solutions and then were stored at − 20 °C. Stock solutions were diluted at the time of each application.

Female spiders in the penultimate nymphal stage (day 35 after leaving a cocoon) were randomly divided into three main groups. The division criteria were the type of injected/applied compound and their dose per animal.

20-hydroxyecdysone was injected into the abdomen of spider anesthetized on ice at the doses: (i) 10 ng, (ii) 100 ng and (iii) 200 ng in volume 0.4 μl per animal. Fenoxycarb and precocene I were applied topically on the dorsal part of the abdomen on each anesthetized spider on ice at a volume of 1 μl per animal. Fenoxycarb was used at the doses: (i) 1 ng, (ii) 10 ng and (iii) 100 ng per spider and precocene I in doses: (i) 10 ng, (ii) 100 ng and (iii) 1000 ng per spider.

At the same time, two control groups for each experimental group: (i) without treatment and (ii) injected with the Ringer solution for the 20-hydroxyecdysone groups or a topical application of acetone for the precocene I and fenoxycarb groups were created. Volumes of compounds that were used were identical to the corresponding experimental groups. After the injection/topical application, animals were kept under the same conditions as the basic breeding described above.

Tissue was collected at various time points after treatment that corresponded with the important stages of female development: immediately after treatment – day 0 (the penultimate nymphal stage), day 1, day 3 (the last nymphal stage), day 5 (the day that sexual maturity was reached), day 8 (three days after mating) and day 12 after the injection/topical application (the day of the first oviposition).

Vg concentration was measured in the midgut glands, ovaries and hemolymph for all of the time points using ELISA. The Pt*Vg*4 expression level was determined in the midgut glands and ovaries at all of the time points. The number of tissues for one sample depended on the age of females and is presented in Table 2 and Table 3. Method of tissue collection as well as the ELISA and qPCR protocols were identical to those described above. In this stage of the experiment, tissues from 500 female spiders were used for each dose and time point.

Analysis of variance for the level of the Pt*Vg* expression and Vg concentration from various experimental groups was performed using MANOVA using the type of compound that was used, its dose, the type of tissue and the time that had elapsed from the injection/topical applications as the sources of any differences. The linearity of any increase/decrease in the levels of the Pt*Vg*4 and Vg relative to the subsequent tested part of development was measured by determining the *R*^*2*^ value. The Pearson correlation for determining the relationship between levels of the Pt*Vg*4 and Vg in various tissues was used. Any differences between experimental groups at the same time point were determined using Tukey’s multiple comparisons test.

Only the results for the group that was treated with Ringer’s solution/acetone are presented in the graph due to the fact that no statistically significant differences were observed between this group and the control group.

## Abbreviations

20E: 20-hydroxyecdysone
ELISA: Enzyme Linked Immunosorbant Assay
HG: Hindgut
INT: Integument
MG: Midgut glands
NS + NS: The nervous system and neuroendocrine system
OV: Ovaries
*Vg*: The gene encoding the vitellogenin
Vg: Vitellogenin at the protein level
